# Comparative Analysis of the Structure and Pharmacological Properties of Some Piperidines and Host–Guest Complexes of β-Cyclodextrin

**DOI:** 10.3390/molecules29051098

**Published:** 2024-02-29

**Authors:** Ulan Kemelbekov, Vitaly Volynkin, Symbat Zhumakova, Kulpan Orynbassarova, Marina Papezhuk, Valentina Yu

**Affiliations:** 1 South Kazakhstan Medical Academy, 1 Al-Farabi Square, Shymkent 160019, Kazakhstan; kulpan_ok@mail.ru; 2 A.B. Bekturov Institute of Chemical Sciences, 106 Ualikhanov St., Almaty 050010, Kazakhstan; symba_t@mail.ru (S.Z.); yu_vk@mail.ru (V.Y.); 3 Faculty of Chemistry, Kuban State University, Krasnodar 350040, Russia; marina-marina322@mail.ru

**Keywords:** cyclodextrin, piperidine, in silico, ADME, biological activity, anesthesia, acute toxicity

## Abstract

Pain and anesthesia are a problem for all physicians. Scientists from different countries are constantly searching for new anesthetic agents and methods of general anesthesia. In anesthesiology, the role and importance of local anesthesia always remain topical. In the present work, a comparative analysis of the results of pharmacological studies on models of the conduction and terminal anesthesia, as well as acute toxicity studies of the inclusion complex of 1-methyl-4-ethynyl-4-hydroxypiperidine (MEP) with β-cyclodextrin, was carried out. A virtual screening and comparative analysis of pharmacological activity were also performed on a number of the prepared piperidine derivatives and their host–guest complexes of β-cyclodextrin to identify the structure–activity relationship. Various programs were used to study biological activity in silico. For comparative analysis of chemical and pharmacological properties, data from previous works were used. For some piperidine derivatives, new dosage forms were prepared as beta-cyclodextrin host–guest complexes. Some compounds were recognized as promising local anesthetics. Pharmacological studies have shown that KFCD-7 is more active than reference drugs in terms of local anesthetic activity and acute toxicity but is less active than host–guest complexes, based on other piperidines. This fact is in good agreement with the predicted results of biological activity.

## 1. Introduction

Local anesthetics are currently used in almost all areas of practical medicine [1]. The interest in local anesthetics is due to the negative side effects of general anesthesia on the cardiovascular system, central nervous system, gastrointestinal tract, and individual organs. Although a large number of local anesthetic drugs are known, a rather limited number of drugs are used in practice [2].

This is due to the fact that most local anesthetics do not correspond to modern standards and requirements [3]. Thus, they must have a short latent period, a long period of action and high activity, and be non-irritating and low-toxic.

One of the most rational drug design approaches towards pharmacologically active molecules is based on the structural modification of compounds with reported high activity. As we can see from the papers [4,5], some 1-alkoxyalkyl-4-hydroxypiperidine hydrochlorides and previously reported 1-ethoxyethyl analogs have revealed local anesthetic effects [6]. At the same time, as reported previously, the corresponding benzoates were found to be the strongest local anesthetics [7].

It is a well-known fact that there is no clear correlation between the chemical structure of a drug and its biological effects [1]. Thus, minor changes in the structure of a molecule may lead to a complete disappearance or a strong change in the biological activity (e.g., methyl and ethyl alcohol). Modern pharmaceutical research and development is a high-risk investment that typically faces setbacks at various stages of drug development [8]. Because of that, a molecular design based on the use of prediction software has attracted so much attention in recent years [9]. The structure–activity relationship analysis of the known drugs can help predict the chemical structure of new molecules with the desired properties [8].

One of the main reasons for failure in drug research and development is the lack of efficacy and safety, which are substantially correlated with absorption, distribution, metabolism, and excretion (ADME), as well as with toxicity (T) [10]. Therefore, a rapid evaluation of the ADMET parameters is necessary to minimize failures in the drug discovery process. The ADME parameters [11,12] cover pharmacokinetics, which determine whether the intended drug molecule will reach the target protein in the body and how long it will remain in the bloodstream.

Cyclodextrins are widely used in the pharmaceutical industry for transporting and modification of an active substance [13,14]. The formation of inclusion complexes makes it possible to change the properties of the biologically active component in the desired direction, i.e., to increase the bioavailability and resistance to hydrolysis, solubility, and biodegradability of many active substances [15,16]. In order to improve the anesthetic effects and reduce the toxicity of the water-soluble salt forms of piperidine derivatives, we synthesized and studied [7,17,18,19,20,21] the host–guest complexes of some of these compounds with β-cyclodextrin.

In this work, a pharmacological study of terminal anesthesia was conducted, and the acute toxicity of the 1-methyl-4-ethynyl-4-hydroxypiperidin (MEP) inclusion complex with β-cyclodextrin was analyzed. Virtual screening of the pharmacological activity for a number of piperidine derivatives was carried out in order to identify the structure–activity relationship. The results of the virtual screening were compared with their actual pharmacological effects.

## 2. Results and Discussion

**Figure 1:**
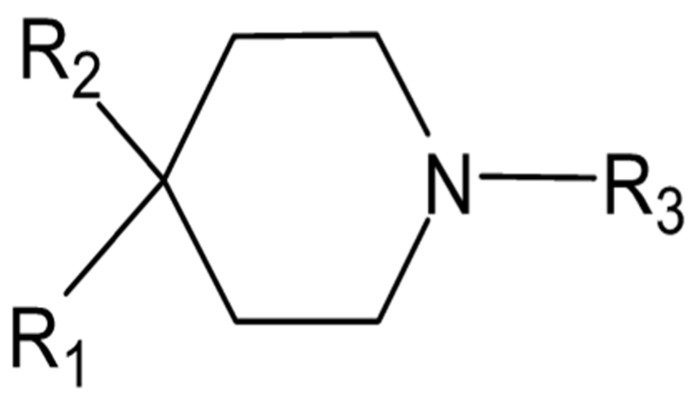
The study compounds (R_1_ = C≡CH, C≡CH=CH_2_, C≡CPh; R_2_ = OCOCH_3_, OCOC_2_H_5_, OCOPh; R_3_ = CH_3_, C_2_H_4_OC_2_H_5_, C_3_H_6_OC_4_H_9_) (see Table 1).

To determine the structure–activity relationship, we used piperidines of the general formula, shown in Figure 1.

### 2.1. In Silico Pharmacology

In drug development, efficient target binding is not only important, but it also ensures oral bioavailability and drug-like properties. In this regard, the study of the physicochemical properties of compounds is crucial for drug development.

The predictive analysis and in silico studies of possible targets, ADME parameters (absorption, distribution, metabolism, and excretion), and compliance with the bioavailability criteria [11,22] were carried out for the studied compounds.

An analysis of the structures for compliance with Lipinski’s rule of five (molecular weights (MW) ≤ 500, cLogP ≤ 5.0, TPSA ≤ 140 Å^2^, number of H-acceptors ≤ 10, H-donors ≤ 5) [23,24] was performed, using the SwissADME software package [25]. Compliance with Lipinski’s rule makes the compounds active drug candidates. The substance is unlikely to become an active drug candidate if Lipinski’s rule is violated even by one parameter.

The analysis of lipophilicity (LogP) is provided in Table 2. Optimal values for LogP (P is the partition coefficient of all forms of the molecule between n-octanol and water) are between 0 and 3. LogP < 0 corresponds to the bad permeability of the lipid bilayer; LogP > 3 indicates poor water solubility [26]. Compounds with high cLogP values may have difficulty in achieving the therapeutic targets due to their lipophilicity, which potentially limits their effectiveness.

The LogP value shows moderately good (0.33) absorption and permeability for the MEP. For EEHP and MEBP, the cLogP values are 1.07 and 2.42, respectively. For the other compounds, the distribution coefficient is significantly higher and ranges from 3.30 to 4.93. More positive cLogP values usually indicate a higher concentration of the compound in the lipid phase.

LogS values (logarithm of water solubility value, expressed in log mol/L) above −4 logmol/L and below 10 µg/mL indicate low solubility. In the range of 10–60 μg/mL, the compounds have moderate solubility. All LogS values higher than 60 µg/mL indicate high solubility [27].

The TPSA parameter for EEHP, MEBP, and MEP has a low value of 23.47 Å^2^ and meets the criteria for oral bioavailability. The MEP compound meets the Lipinski, Egan, and Weber criteria. The Egan filter (Pharmacia filter) is based on the LogP and TPSA parameters. It anticipates drug absorption, depending on the processes involved in the membrane permeability of a small molecule, and considers the molecule drug-like if it has WLOGP ≤ 5.88 and TPSA ≤ 131.6, respectively [28]. The Muegge filter (the Bayer filter) is the independent pharmacophore point filter that separates drug-like and non-drug-like molecules. The Ghose filter (Amgen) describes small molecules based on their physicochemical properties and the existence of functional groups and substructures [28]. EEHP only fails to meet the Muegge criteria due to its low molecular weight. BBB·HCl has failed to meet the Ghose criteria because the calculation was carried out for a hydrochloride form. The remaining compounds correspond to all the criteria provided in Table 3.

All compounds have shown favorable bioavailability values (0.55). This indicates good suitability for oral drug administration and implies achieving a therapeutic result at lower concentrations.

**Figure 2:**
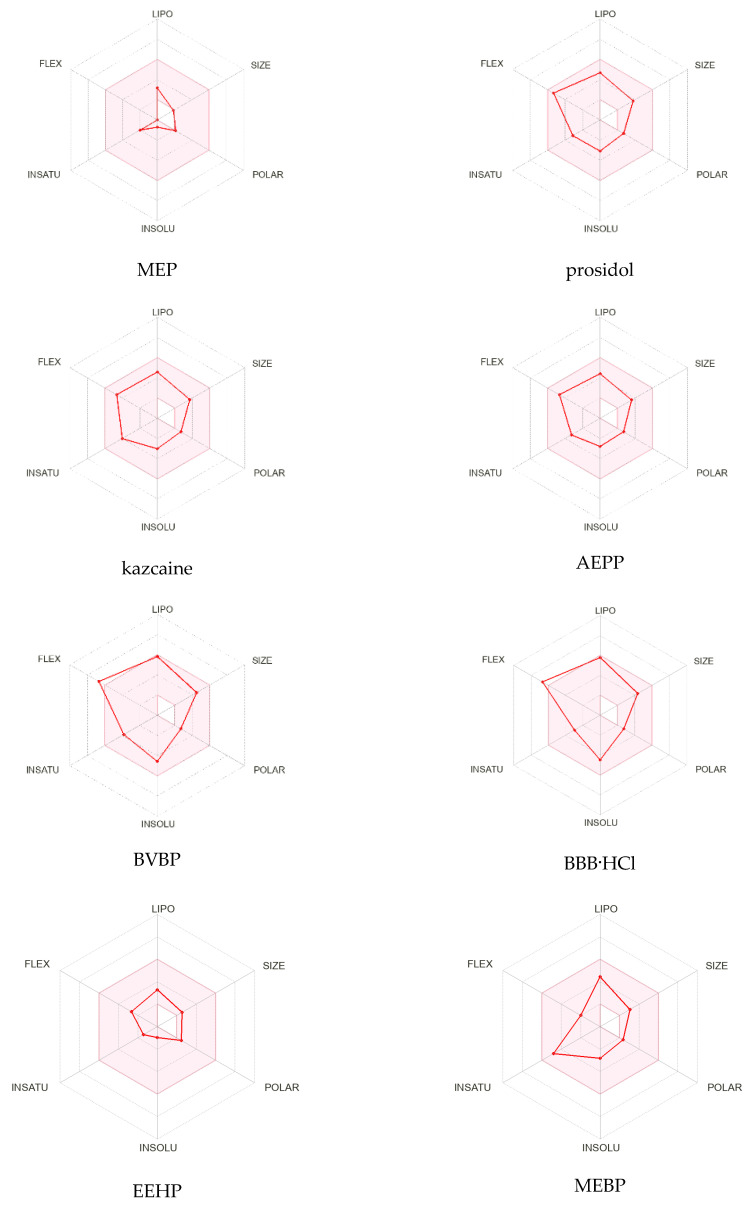
The bioavailability radar of the studied compounds based on the physicochemical indices ideal for oral bioavailability.

The radar diagrams (Figure 2) show the distribution of the physicochemical properties of the compounds: lipophilicity (LIPO), size (SIZE), polarity (POLAR), solubility (INSOLU), saturation (INSATU), elasticity (FLEX), presence of donors (nHD), and proton acceptors (nHA). The pink area represents the optimal range for each property (lipophilicity: XLOGP3 −0.7 to +5.0, size: molecular weight 150 to 500 g/mol, polarity: TPSA 20 to 130 Å^2^, solubility: log S not above 6, saturation: the fraction of carbons in sp^3^ hybridization is at least 0.25 and flexibility no more than nine rotating bonds) [25]. The analyses of the diagrams show that prosidol, kazcaine, and AEPP have the best distribution of parameters, though all the compounds, in principle, meet the requirements for a medicinal substance. BVBP and BBB have a slight excess in the FLEX parameter, and for MEP, the size, polarity, and flexibility indicators are at the lower limit.

To predict possible biological effects, the open software products PASS Online, AntiBac-Pred, and AntiFun Pred [29,30,31] were used. Here, and below, the score function F = Pa − Pi is used, which is the difference in the probabilities that a substance will be active (Pa) or inactive (Pi) for the corresponding biological activity.

In Table 4, the results for MEP are provided (for F > 0.1). Based on these data, the most probable biological activity of MEP is the suppression of ovulation; there is also a very high probability of its influence on the hormones responsible for reproductive functions. The substance can be used as an anticonvulsant. The other activities (anesthetic, anabolic, nootropic, antidepressant, analgesic, and muscle relaxant) have a rather low probability. Comparative data on the major types of activity for all the substances under consideration are provided in Table 5. The results are provided for the substances in the form of bases since the calculation programs, in most cases, cannot work with the substances in the form of salts and complex compounds (including inclusion complexes).

Possible protein targets (for *Homo sapiens*) were evaluated using the Swiss Target Prediction service. The results are shown in Table 6. The score for each target is called “confidence”, which is the difference between probabilities of chemical compounds interacting and not interacting with a particular target. Higher confidence means a higher chance of a positive prediction being true. The first 5–6 results are listed and the rest are provided in the Supplementary Materials. The probabilities for MEP are very low, but we can conclude that the substance may affect mechanisms that occur in the central nervous system.

The PASS Targets program provides a slightly different prediction of possible molecular targets. It is advisable to consider results with a confidence value greater than 0.5. Table 7 shows the values greater than 0.5 for MEP, EEHP, and MEBP and greater than 0.25 for the remaining compounds. The full list is presented in Table S1.

According to Table 7, MEP has the largest number of possible targets with a confidence value greater than 0.5. It looks most similar to kazcaine according to the list of possible targets, though the character of the data obtained (a large number of targets and high probability values) should rather be considered an anomaly. The substances MEP, EEHP, and MEBP actively bind to protein kinases.

In silico prediction of acute toxicity values (LD50) for rats for four types of administration (oral, intravenous, intraperitoneal, subcutaneous, and inhalation) was carried out using the GUSAR program [32]. This program compares the structure of a substance with structures from the SYMYX MDL toxicity database. In order to assess which of these drugs best corresponds to the optimal characteristics required for an ideal drug, the acute toxicity parameter LD50 (known as the “lethal dose, 50%” or oral acute dose for rats) was calculated. High toxicity was indicated by values of 1–50 mg/kg; average toxicity was in the range of 51–500 mg/kg. Low toxicity values were 501–5000 mg/kg [33]. The GUSAR program could not calculate data for BBB HCl in the form of either hydrochloride (which is expected) or a base.

The acute toxic class is provided according to the OECD. Low concentrations of the substance reduce the risk of side effects and toxicity. Analyzing the data in Table 8, it can be argued that the acute toxicity values of the compounds exceed the values of the average toxicity range for the compounds prosidol, AEPP, and BVBP. MEP showed a fairly low predicted toxicity risk for intraperitoneal, intravenous, and subcutaneous administration but higher toxicity for all routes of administration compared to the other study drugs.

**Figure 3:**
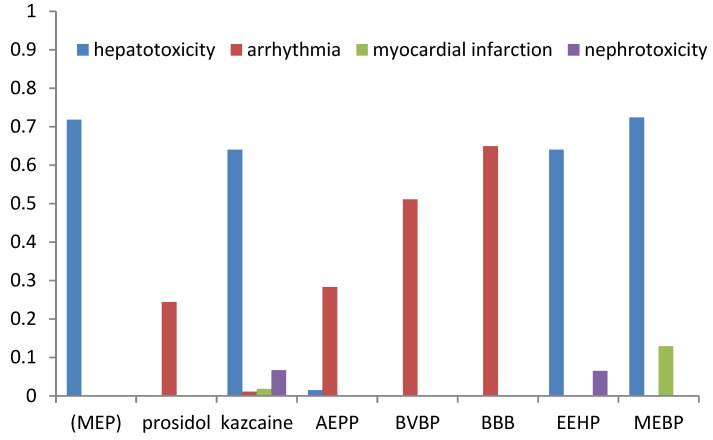
The probability of adverse effects for the compounds under study.

The prognosis of adverse effects (arrhythmia, heart failure, hepatotoxicity, myocardial infarction, and nephrotoxicity) was made using ADVER Pred [34]. The results are shown in Table 9 and Figure 3.

The compounds may exhibit side effects such as arrhythmia (prosidol, kazcaine, AEPP, BVBP, and BBB), hepatotoxicity (MEP, kazcaine, AEPP, EEHP, and MEBP), myocardial infarction (kazkain and MEBP), and nephrotoxicity (MEBP and EEHP). Kazcaine was predicted to cause the highest number of adverse effects compared to the other compounds. However, their probability, excluding hepatotoxicity, was low. The calculated results also indicate a high probability of hepatotoxicity for MEP. For most compounds, a high probability of arrhythmia was predicted as an adverse effect. In order to improve the bioavailability parameters and reduce the toxic side effects, it is advisable to use active compounds in the form of inclusion complexes with cyclodextrin.

### 2.2. Host–Guest Complexes with β-Cyclodextrin

The severity of adverse effects, such as hepatotoxicity and nephrotoxicity, can be reduced using drug inclusion complexes with β-cyclodextrin. Cyclodextrins usually improve the solubility of guest molecules in water, significantly reduce their toxicity, and increase the period of action due to the slow dissociation of the inclusion complex in the body.

Usually, the drugs are used not in a pure form but in a so-called “dosage form”. For example, water-soluble drugs are used in the form of isotonic solutions containing a local anesthetic, while fat-soluble drugs are administered subcutaneously in the form of an oil solution, from which the drug slowly passes into the interstitial fluid.

Earlier, piperidines have been often used as water-soluble salt forms, such as hydrochlorides, to prepare useful dosage forms.

However, along with a high anesthetic effect, such dosage forms also have significant toxicity. Therefore, the preparation of new dosage forms with minimal adverse effects is an actual problem.

The preparation of new dosage forms based on inclusion (host–guest) complexes of cyclodextrins seems to be a promising solution to the problem.

Inclusion complexes are effective as delivery tools. With the conventional type of administration, only nearly one-tenth of the drug molecules can reach the site of application (nerves, tumors, etc.). When the drug is delivered in the form of an inclusion complex and released directly near the site of application, the effective local concentration is increased. Therefore, less amount of drug is required, which can also reduce overall toxicity.

In our previous works [7,13,14,15,16,17,18,19,20,21], we reported the preparation of host–guest complexes of the above piperidines with β-CD and studied their structure (Table 10). All the compounds except MEP formed inclusion complexes with a guest–host ratio of 1:2. For MEP, the 1:1 complex was isolated, which is most likely due to the smaller size of the guest molecule.

The structures of inclusion complexes were studied by NMR during their complex formation in the solutions as well as by X-ray diffraction in their crystalline form. Due to the flexibility of the piperidine ring, piperidines can exist in two main conformations. In inclusion complexes, they can either remain in their starting conformation (for example, BBB) or have a different conformation compared to their free form (kazcaine and prosidol). In addition, in a solution (CDCl_3_ and D_2_O), BBB-HCl exists as two isomers in a 2:1 ratio with different orientations of benzoyloxy groups: 1e-(3-n-butoxypropyl)-4a-benzoyloxypiperidine hydrochloride and 1e-(3-n-butoxypropyl)-4e-benzoyloxypiperidine hydrochloride. BBB-HCl forms inclusion complexes with β-CD with a stoichiometry of 2 β-CD:1 BBB-HCl. The same conformation also exists in the inclusion complex isolated in the solid form.

**Figure 4:**
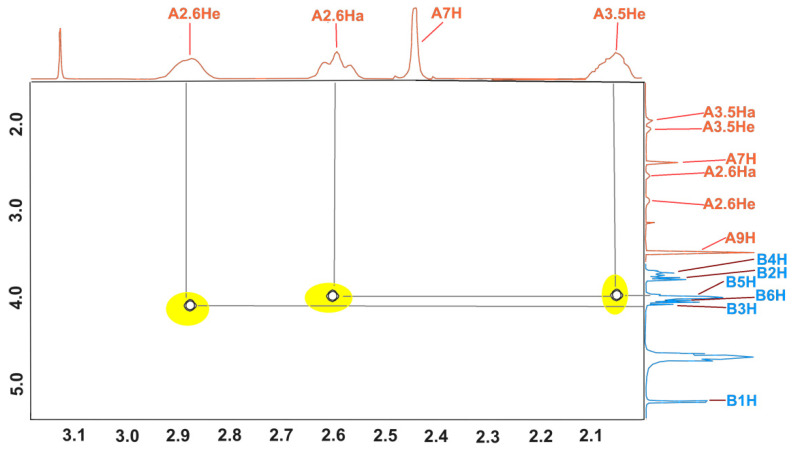
ROESY spectrum of the MEP–β-CD complex.

The structure of the inclusion complex of β-CD with MEP (KFCD-7) was studied using NMR and X-ray diffraction [21]. Below (Figure 4), the expansion from the ROESY NMR spectrum in addition to the data published earlier are shown. The cross peaks between inner (3 and 5) protons of β-CD and 2 and 6 protons of the piperidine ring clearly show that the structure of the MEP:β-CD complex in the solution corresponds to the one obtained from the X-ray data in the solid state.

An analysis of the predicted biological activity shows that MEP, as well as its β-CD inclusion complex, should be significantly different in biological activity from the other piperidine derivatives and their inclusion complexes. Because of that, we conducted a pharmacological study of KFCD-7 and compared its acute toxicity, infiltration, and conduction anesthesia with the data for previously obtained piperidine derivatives and reference drugs.

### 2.3. Pharmacological Study

#### 2.3.1. Infiltration Anesthesia

The test was performed using the Bulbring–Wade method. All the compounds were tested as 0.5% aqua solutions. The results are summarized in Table 11.

As we can see from Table 11, all the drugs have an anesthetic effect that exceeds both novocaine and lidocaine. KFCD-7 shows a slightly longer duration of complete anesthesia than lidocaine, higher than trimecaine in terms of the anesthesia index (35.4 ± 1.3) and duration of complete anesthesia, but less in total duration of anesthesia. The other piperidine derivatives revealed the best values for all parameters of the infiltration anesthesia.

The only exception is kazcaine which has a duration of complete anesthesia comparable to lidocaine but a higher total duration of anesthesia. The formation of an inclusion complex significantly (two times) increases the duration of complete anesthesia up to the KFCD-6 value.

For BVBP and BBB-HCl, the formation of an inclusion complex does not improve their local anesthetic activity. In the case of BBB-HCl, the formation of an inclusion complex significantly (more than two times) reduces the duration of complete anesthesia, while for BVBP, this effect is not so dramatic. The duration of complete anesthesia increases in the following order: procaine < lidocaine < kazcaine < trimecaine < KFCD-7 < BBB-HCl:β-CD < KFCD-4 < KFCD-6 < kazcaine:β-CD < BVBP < BBB-HCl. Total duration of effect: procaine < lidocaine < KFCD-7 < trimecaine < KFCD-4 < kazcaine < KFCD-6 < BVBP < BBB-HCl:β-CD < BBB-HCl < kazcaine:β-CD.

**Figure 5:**
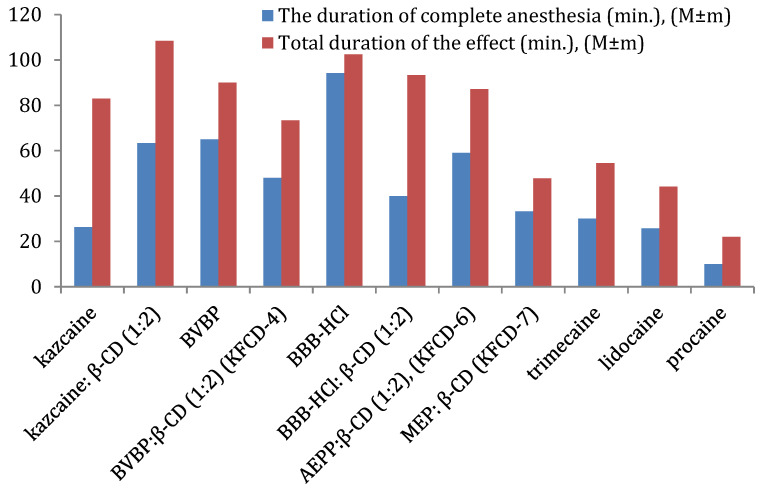
The comparison of local anesthetic activity of the compounds and reference drugs for the infiltration anesthesia, using the Bulbring–Wade method (concentration 0.5%).

Overall (Figure 5), the kazcaine:β-CD inclusion complex is comparable to BVBP, while KFCD-6 has a slightly shorter duration of complete anesthesia. KFCD-6 and KFCD-4 are better than procaine by 5.9 and 4.8 times, lidocaine by 2.3 and 1.9 times, and trimecaine by 2.0 and 1.6 times, respectively. They have a longer total duration of effect than trimecaine by approximately 2 and 1.7 times, lidocaine by 2.3 and 1.3 times, and procaine by 4.0 and 3.3 times, respectively (statistically significant at *p* < 0.05).

#### 2.3.2. Conduction Anesthesia

A modified “tail flick” method was used in the study of conduction anesthesia [36]. It was developed at the Department of Pharmacology of the St. Petersburg Medical University, named after Academician I.P. Pavlov. The principle of the method is to determine the latent period of tail withdrawal during the thermal exposure of its middle part with a focused beam of light from an optoelectronic analgesimeter TF-003 before and after anesthesia. The intensity of the thermal nociceptive stimulus is adjusted so that initial tail flick responses occur with a latency ranging from 3 to 6 s.

The activity of compounds and reference drugs for the conduction anesthesia wasstudied in 1% solutions. The following parameters were determined: the rate of onset of anesthesia, the duration of the complete anesthesia, and the total duration of effect.

**Figure 6:**
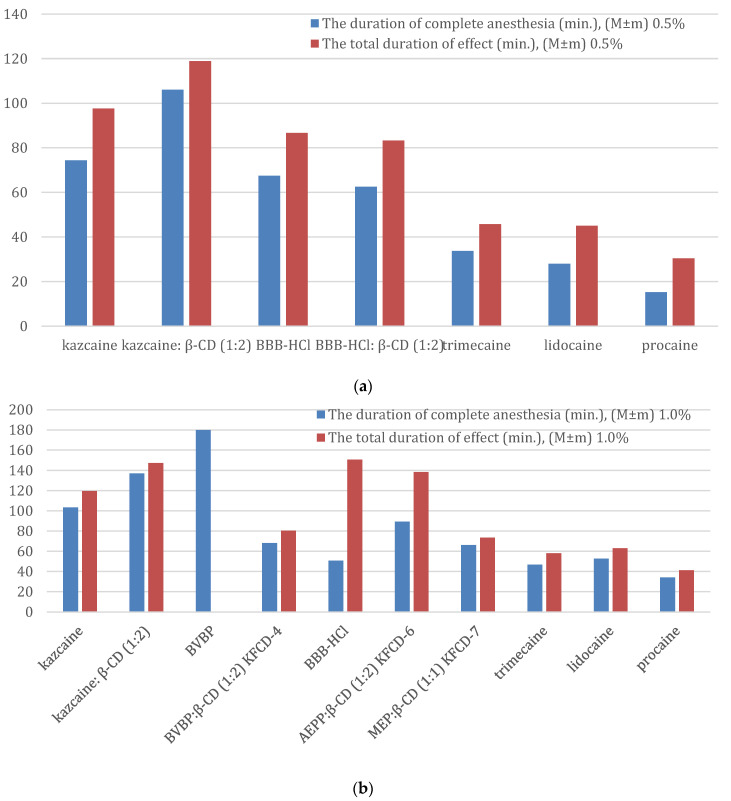
(**a**) The comparison of the duration of the complete anesthesia and the total duration of effect for the conduction anesthesia (0.5%). (**b**) The comparison of the duration of the complete anesthesia and the total duration of effect for the conduction anesthesia (1.0%).

The results are shown in Table 12. A comparison of the duration of the complete anesthesia and the total duration of effect is shown in Figure 6a,b.

As can be seen from Table 12, all the complexes have an apparent local anesthetic effect, and the rate of anesthesia induction is comparable in all cases.

The duration of complete anesthesia (0.5%): procaine < lidocaine < trimecaine < BBB-HCl:β-CD < BBB-HCl < kazcaine < kazcaine:β-CD

The total duration of effect (0.5%): procaine < lidocaine < trimecaine < BBB-HCl:β-CD < BBB-HCl < kazcaine < kazcaine: β-CD.

The duration of complete anesthesia (1%): procaine < trimecaine < BBB-HCl < lidocaine < KFCD-4 < KFCD-6 < kazcaine < kazcaine:β-CD < BVBP

The total duration of effect (1%): procaine < trimecaine < lidocaine < KFCD-7 < KFCD-4 << kazcaine < KFCD-6 < kazcaine: β-CD ≈ BBB-HCl.

KFCD-7 outperformed all three reference anesthetics for the duration of anesthesia and total anesthetic effect and acted like KFCD-4 (Table 12).

At the above-mentioned concentrations, KFCD-4 and KFCD-7 exceeded procaine for the duration of complete anesthesia by 2 and 1.9 times, trimecaine by 1.3 and 1.4 times, respectively, and acted slightly longer than lidocaine. These solutions also exceeded novocaine and trimecaine in the total duration of a local anesthetic effect (approximately 1.9 and 1.4 times, respectively) and slightly exceeded the effect of lidocaine.

As for the other drugs under consideration, the best result of conduction anesthesia at a 1% solution was exhibited by BVBP, almost three times longer than its complex with CD (KFCD-4); that is, the same picture was observed as for infiltration anesthesia.

The duration of complete anesthesia for KFCD-6 was 89.4 ± 13.4 min, 46.9 ± 8.1 min for trimecaine, 52.7 ± 6.2 for lidocaine, and 34.2 ± 6.9 min for procaine. Thus, the KFCD-6 complex exceeded procaine by 2.3 times, lidocaine by 1.2 times, and trimecaine by 1.7 times (statistically significant at *p* < 0.001). When comparing the total duration of effect, the KFCD-6 reliably (*p* < 0.001) exceeded trimecaine by 2.3 times, lidocaine by 2.2 times, and procaine by 3.4 times, respectively.

Kazcaine initially had a good activity (three times better than procaine), and its complex with CD improved the duration of complete anesthesia and the total duration of a local anesthetic effect (but not so dramatically, approximately 30%).

The results for BBB-HCl look interesting. Similar to the infiltration anesthesia, the formation of the complex did not provide an increase in the activity for a 0.5% concentration. However, what is unexpected is that for the 1% concentration, the duration of complete anesthesia was shorter, but the total duration of anesthesia was longer than for the 0.5% concentration. This may probably be due to the different measurement methods used for the 1% solution.

#### 2.3.3. Acute Toxicity

**Figure 7:**
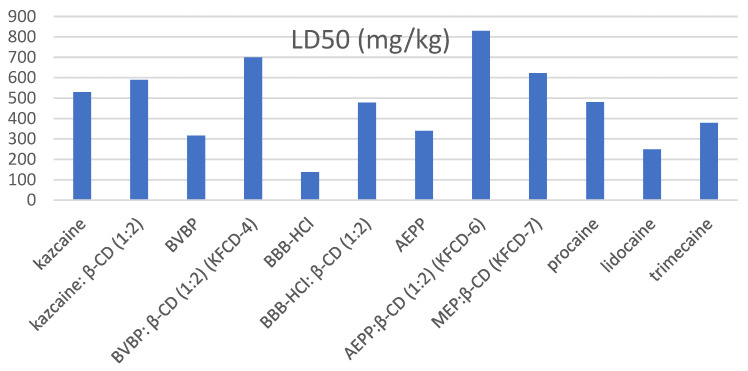
The acute toxicity of the compounds under study and the reference drugs.

Behavior changes, reflector breath excitability, rate of development and mitigation of external poisoning symptoms, and mortality (LD50) were registered (Figure 7).

The toxic reactions were of the same character for KFCD-4, KFCD-6, and KFCD-7. The higher the dose, the faster poisoning was evident. The phenomena of intoxication began to develop after 20–30 min. The initial stage started with general oppression and resulted in a deferred response, absence of reflex to exogenous irritants, and dyspnea, which later developed into a short period of motional excitation, followed by muscular twitching and clonic–tonic spasms. Mice assumed a lateral position and their breathing became slower and irregular. Death was caused by primary respiratory standstill 30–90 min after injection. The surviving mice recovered from stagnation in 2–2.5 h and were as active as the untreated mice by the end of the first day.

An analysis of the data obtained for the entire group of the drugs under consideration (Table 13) showed that the formation of the inclusion complexes significantly decreases the acute toxicity of substances. The resulting inclusion complexes of piperidine derivatives with β-CD were significantly less toxic than the guests themselves.

The KFCD-6 compound turned out to be the most active and less toxic than procaine by 1.7 times, lidocaine by 3.3 times, and trimecaine by 2.2 times in all experiments (Table 13). KFCD-7 was less toxic than the reference anesthetics.

The most toxic was BBB-HCl, but in the form of an inclusion complex, its toxicity dropped by more than three times and became comparable to procaine. The formation of an inclusion complex reduced the toxicity of AEPP by 2.4 times and BVBP by 2.2 times. The toxicity of kazcaine (which is slightly less than procaine) remained virtually unchanged upon the formation of the inclusion complex, becoming comparable to KFCD-7.

#### 2.3.4. Terminal Anesthesia 

The comparison of the activity of the tested compounds with the reference anesthetic, dicaine, was carried out using the Rainier indices, duration of the complete anesthesia, and total duration of effect.

All the studied compounds were tested in 1% and 3% solutions. The experimental results showed that the compounds KFCD-4, KFCD-6, and KFCD-7 in all tested concentrations were significantly inferior both in strength (the Ragnier index) and in the duration of the local anesthetic effect to dicaine and in all concentrations they did not show irritating effects.

At the same time, the formation of inclusion complexes does not always lead to higher activity and depends both on the characteristics of the “guest” and on the type of anesthesia. The most effective in this sense was the inclusion complex of cyclodextrin with 1-(2-ethoxyethyl)-4-ethynyl-4-benzoyloxypiperidine, which is two times better for infiltration anesthesia and 30% better for conduction anesthesia than its salt form (1-(hydrochloride 2-ethoxyethyl)-4-ethynyl-4-benzoyloxypiperidine).

According to the literature, the extension of the alkyl chain at the N atom of the piperidine derivative to the ethoxyethyl substituent leads to the anesthesia index exceeding trimecaine by 1.5 times, lidocaine by 5.1, procaine by 5.3 times, including piperidine derivatives with butoxypropyl substituent. The EC50 value for conduction anesthesia of 1-(3-n-butoxypropyl)-4-benzoyloxypiperidine hydrochloride exceeds the ethoxyethyl homologue by 140 times, and the reference drugs pyromecaine, trimecaine, and procaine by 270, 446, and 670 times, respectively [38].

This pattern is also confirmed by good results for BVBP and BBB-HCl. Elongation of the radical at the nitrogen atom of the piperidine ring from ethoxyethyl to butoxypropyl led to a significant increase in activity during infiltration, especially during the conduction of anesthesia. However, these same drugs have the highest toxicity among those considered. The formation of inclusion complexes leads to a significant reduction in toxicity (comparable to trimecaine) but, at the same time, to a significant reduction in the anesthesia time.

## 3. Materials and Methods

The following programs were used to study biological activity in silico. The physicochemical and pharmacokinetic properties, including the physicochemical parameters, lipophilicity, absorption, distribution, metabolism, and drug affinity, i.e., the ADME profiles [25], were analyzed on the SwissADME web server (http://www.swissadme.ch/index.php accessed on 7 July 2023). The drug similarity of compounds based on Lipinski’s rule of five was also predicted using the SwissADME web server, and toxicity analysis was carried out with the GUSAR program (https://www.way2drug.com/Gusar/ accessed on 7 July 2023) [32]. To predict possible biological effects, PASS Online open-source software was used [29] (https://www.way2drug.com/PassOnline/ accessed on 12 August 2023). The prognosis of adverse effects was made using ADVER Pred [34] (http://www.way2drug.com/adverpred/ accessed on 12 August 2023). Possible protein targets were evaluated using the Swiss Target Prediction service [39] (http://swisstargetprediction.ch/ accessed on 10 June 2023) and the PASS Targets program [40] (https://www.way2drug.com/passtargets/ accessed on 10 June 2023).

The infiltration anesthesia test was performed with the Bulbring–Wade method [41]. The studies were conducted on male guinea pigs with average masses of 200–250 g. The samples of isotonic solutions of the studied compounds and reference drugs were injected intradermally (0.2 mL) in the back of each animal at four points (vertices of the square with a side of 3 cm) after hair removal. The local anesthetic activity was evaluated six to eight times for each of the selected concentrations. Sensitivity at the injection site was determined by the touch of a blunt injection needle for a series of six touches every 5 min until full recovery.

The depth of anesthesia, expressed as the “anesthesia index” (average of 6 experiments, maximum index-36), the duration of complete anesthesia, and the total duration of the anesthetic effect were determined. The activity of the compounds was compared with the reference drugs, trimecaine, lidocaine, and novocaine, in corresponding concentrations.

The study of conduction anesthesia was carried out using a modified “tail flick” method in rats [36]. It allows one to determine the speed of onset of anesthesia, its depth, the duration of the complete anesthesia, and the total duration of the anesthetic effect of the drug. The study was carried out on outbred white male rats weighing 200–250 g. To study the conduction anesthesia, a solution of a compound or drug (0.5 mL) was injected under the skin of the tail into the area where the thermal effect was applied. The animals in the control group were injected with a saline solution in the same way and same volume. Irritation was applied 1 cm distal from the injection. The first test was carried out 5 min after injection; subsequent tests were carried out every 10 min until the threshold values were completely restored. Doubling of the latent period was taken as complete anesthesia.

Acute toxicity was determined after a single subcutaneous injection of the studied compound and reference drugs in mice (6–8 outbreed albino mice weighing 17.0–22.0 g).

The symptoms of poisoning, speed of onset, severity of regression, and mortality rate were recorded. The animals that survived the first 24 h were monitored in terms of their behavior and full recovery of appetite. The lethal dose (LD50) was calculated using the Miller and Tainter method [42].

All the data obtained were statistically treated.

## 4. Conclusions

The analysis of the data obtained for the entire group of drugs under consideration shows that the formation of inclusion complexes significantly decreases the acute toxicity of substances.

Based on the results obtained, we can conclude that the inclusion complexes of piperidine derivatives under study are low-toxic local anesthetics, for which further research and development as pharmaceuticals are advisable. Of these, the inclusion complexes of kazcaine and AEPP can be considered the most promising. Moreover, recently obtained fluorine derivatives of kazcaine have shown unexpected antimicrobial activity [43,44].

The pharmacological study results determined that, in terms of local anesthetic activity and acute toxicity, KFCD-7 exceeded all the drugs in comparison but is inferior to all other considered inclusion complexes of piperidine derivatives. The predicted biological activity confirmed the results of the pharmacological study and has shown that both MEP and its complex KFCD-7 are promising molecules for further studies of anticonvulsant effects and effects on reproductive functions.

## Figures and Tables

**Table 1:** The compounds used in the present work.

	Formula	Code	Name	R_1_	R_2_	R_3_	Ref.
1	C_18_H_23_NO_3_	kazcaine	1-(2-ethoxyethyl)-4-ethynyl-4-benzoyl oxypiperidine	-C≡CH	-(OCO)-C_6_H_5_	-(CH_2_)_2_-O-C_2_H_5_	[7]
2	C_18_H_27_NO_3_	prosidol	1-(2-ethoxyethyl)-4-phenyl-4-propionyl oxypiperidine	-C_6_H_5_	-(OCO)-C_2_H_5_	-(CH_2_)_2_-O-C_2_H_5_	[8]
3	C_19_H_29_NO_3_·HCl	BBB·HCl	1-(3-*n*-butoxypropyl)-4-benzoyl oxypiperidine hydrochloride	-H	-(OCO)-C_6_H_5_	-(CH_2_)_3_-O-C_4_H_9_	[9]
4	C_23_H_31_NO_3_	BVBP	1-(3-*n*-butoxypropyl)-4-vinylacetilene-4-benzoyloxypiperidine	-C≡C-CH≡CH_2_	-(OCO)-C_6_H_5_	-(CH_2_)_3_-O-C_4_H_9_	[10]
5	C_17_H_25_NO_3_	AEPP	4-acetoxy-1-(2-ethoxyethyl)-4-phenyl piperidine	-C_6_H_5_	-(OCO)-CH_3_	-(CH_2_)_2_-O-C_2_H_5_	[11]
6	C_8_H_13_NO	MEP	1-methyl-4-ethynyl-4-hydroxypiperidine	-C≡CH	-OH	-CH_3_	[12]
7	C_11_H_19_NO_2_	EEHP	1-(2-ethoxyethyl)-4-ethynyl-4-hydroxypiperidine	-C≡CH	-OH	-(CH_2_)_2_-O-C_2_H_5_	[15]
8	C_15_H_17_NO_2_	MEBP	1-methyl-4-ethynyl-4- benzoyl-oxypiperidine	-C≡CH	-(OCO)-C_6_H_5_	-CH_3_	[14]

**Table 2:** Physical and chemical parameters of the studied compounds.

Compounds	Physical and Chemical Parameters					
	cLogP	log*S*	MW, g/mol	TPSA, Å^2^	Bioavailability	Synthetic Accessibility
MEP	0.33	−0.73	139.19	23.47	0.55	1.88
prosidol	3.5	−3.1	305.41	38.77	0.55	2.48
kazcaine	3.73	−3.02	301.38	38.77	0.55	2.76
AEPP	3.30	−2.79	291.39	38.77	0.55	2.32
BVBP	4.93	−4.57	369.50	38.77	0.55	3.76
BBB·HCl	1.29	−4.49	355.90	39.97	0.55	3.33
EEHP	1.07	−0.96	197.27	32.70	0.55	2.30
MEBP	2.42	−2.83	243.30	29.54	0.55	2.33

**Table 3:** The bioavailability criteria for the compounds under study.

Compounds	Criteria				
	Lipinski	Ghose	Veber	Egan	Muegge *
MEP	Yes	No: MW < 160	Yes	Yes	No: MW < 200
prosidol	Yes	Yes	Yes	Yes	Yes
kazcaine	Yes	Yes	Yes	Yes	Yes
AEPP	Yes	Yes	Yes	Yes	Yes
BVBP	Yes	Yes	Yes	Yes	Yes
BBB·HCl	Yes	No: WLOGP < −0.4	Yes	Yes	Yes
EEHP	Yes	Yes	Yes	Yes	No: MW < 200
MEBP	Yes	Yes	Yes	Yes	Yes

* Muegge: MW between 200 and 600 Da, XLogP −2 to 5, TPSA less than 150, number of rings less than 7, number of carbon less than 4, and number of heteroatoms less than 1.

**Table 4:** The predicted biological activity for 1-methyl-4-ethynyl-4-hydroxypiperidine.

Pa	Pi	F	Biological Activity
0.841	0.003	0.838	Ovulation inhibitor
0.690	0.010	0.680	Anticonvulsant
0.678	0.004	0.674	Gonadotropin antagonist
0.673	0.006	0.667	Antiosteoporotic
0.672	0.012	0.660	Antisecretoric
0.581	0.016	0.565	Neurotransmitter antagonist
0.560	0.006	0.554	Dementia treatment
0.611	0.086	0.525	Testosterone 17beta-dehydrogenase (NADP+) inhibitor
0.481	0.043	0.438	Antihypoxic
0.423	0.049	0.374	Analeptic
0.396	0.023	0.373	Antialcoholic
0.371	0.007	0.364	Estrogen agonist
0.393	0.030	0.363	Antiparkinsonian
0.437	0.083	0.354	Antiviral (Picornavirus)
0.384	0.034	0.350	Skeletal muscle relaxant
0.353	0.016	0.337	Antiperistaltic
0.351	0.026	0.325	Antitussive
0.323	0.002	0.321	Estradiol 17 beta dehydrogenase stimulant
0.407	0.101	0.306	Alopecia treatment
0.293	0.008	0.285	Contraceptive female
0.309	0.031	0.278	Antiparkinsonian, tremor relieving
0.287	0.027	0.260	Antinaupathic
0.265	0.030	0.235	Antidepressant, Imipramin-like
0.337	0.104	0.233	Analgesic
0.290	0.059	0.231	Cardiovascular analeptic
0.277	0.064	0.213	Antiparasitic
0.237	0.025	0.212	Antihypotensive
0.398	0.186	0.212	Antiischemic. cerebral
0.273	0.068	0.205	Muscle relaxant
0.209	0.003	0.206	Progesterone agonist
0.320	0.116	0.204	Antiseborrheic
0.273	0.071	0.202	Antiparkinsonian, rigidity relieving
0.317	0.129	0.188	Antipruritic, allergic
0.247	0.061	0.186	Sclerosant
0.401	0.215	0.186	Nootropic
0.315	0.141	0.174	Vasoprotector
0.175	0.012	0.163	Female sexual dysfunction treatment
0.180	0.021	0.159	Estrogen antagonist
0.352	0.197	0.155	Antineurotic
0.262	0.120	0.142	Antiviral (Herpes)
0.153	0.014	0.139	Anabolic
0.203	0.068	0.135	Anesthetic
0.274	0.144	0.130	Antipruritic
0.137	0.016	0.121	Androgen antagonist
0.202	0.091	0.111	Diuretic

**Table 5:** A summary of the predicted biological effects for the studied compounds.

Biological Activity	F									
	MEP	Prosidol	Kazcaine	AEPP	BVBP	BBB	EEHP	MEBP	Lidocaine	Procaine
Anesthetic	0.135	0.752	0.707	0.758	0.737	0.893	0.41	0.427	0.788	0.923
Anesthetic local	-	0.732	0.612	0.733	0.730	0.897	0.327	0.352	0.761	0.914
Analgesic	0.233	0.589	-	0.553	-	-	-		0.053	0.003
Spasmolytic	-	0.576	0.570	0.673	0.442	0.828	0.358	0.367	0.470	0.749
Spasmolytic, urinary	-	0.604	0.670	0.614	0.397	0.672	0.616	0.447	0.722	0.804
Spasmolytic, Papaverin-like	-	0.513	0.685	0.452	0.574	0.839	-	-	0.224	0.786
Anticonvulsant	0.680	0.542	0.526	0.511	0.082	-	0.648	0.562	0.608	0.726
Antidepressant, Imipramin-like	0.235	-	-	-	-	-	-	-	0.084	0.197
Skeletal muscle relaxant	-	0.327	0.272	0.186	0.067	-	-	-	0.334	0.684

**Table 6:** The summary of the most probable macromolecular targets for the studied compounds study (SwissTargetPrediction).

Protein	Confidence	CHEMBL ID
MEP		
Phenylethanolamine N-methyltransferase	0.033970689612	CHEMBL4617
Aminopeptidase N	0.033970689612	CHEMBL1907
prosidol		
Dopamine D3 receptor	0.127302569502	CHEMBL234
Vesicular acetylcholine transporter	0.127302569502	CHEMBL4767
Sigma opioid receptor	0.119403562123	CHEMBL287
Dopamine D2 receptor	0.119403562123	CHEMBL217
Mu opioid receptor	0.119403562123	CHEMBL233
Serotonin 1a (5-HT1a) receptor	0.119403562123	CHEMBL214
kazcaine		
Muscarinic acetylcholine receptor M4	0.11150186548	CHEMBL1821
Muscarinic acetylcholine receptor M2	0.11150186548	CHEMBL211
Muscarinic acetylcholine receptor M1	0.11150186548	CHEMBL216
Muscarinic acetylcholine receptor M3	0.11150186548	CHEMBL245
Dual specificity mitogen-activated protein kinase kinase 1	0.11150186548	CHEMBL3587
AEPP		
Vesicular acetylcholine transporter	0.122581769115	CHEMBL4767
Dopamine D3 receptor	0.114337558605	CHEMBL234
Serotonin 1a (5-HT1a) receptor	0.106099949133	CHEMBL214
Dopamine transporter	0.106099949133	CHEMBL238
Serotonin transporter	0.106099949133	CHEMBL228
BVBP		
Butyrylcholine sterase	0.113285953487	CHEMBL1914
Cathepsin D	0.113285953487	CHEMBL2581
Beta-secretase 1	0.113285953487	CHEMBL4822
Beta secretase 2	0.113285953487	CHEMBL2525
Melanin-concentrating hormone receptor 1	0.113285953487	CHEMBL344
Dopamine transporter	0.113285953487	CHEMBL238
BBB		
Butyrylcholine sterase	0.259910103952	CHEMBL1914
Dopamine transporter (by homology)	0.234886157898	CHEMBL238
Neuronal acetylcholine receptor protein alpha-7 subunit	0.151564691457	CHEMBL2492
Dopamine D3 receptor	0.118277084772	CHEMBL234
Serotonin transporter	0.109945769839	CHEMBL228
Norepinephrine transporter	0.109945769839	CHEMBL222
Sigma opioid receptor	0.109945769839	CHEMBL287
EEHP		
Purine nucleoside phosphorylase	0.0312265582077	CHEMBL4338
Dipeptidyl peptidase IV	0.0312265582077	CHEMBL284
Glutathione reductase	0.0312265582077	CHEMBL2755
Histamine H1 receptor	0.0312265582077	CHEMBL231
Adrenergic receptor beta	0.0312265582077	CHEMBL210
Mu opioid receptor	0.0312265582077	CHEMBL233
Delta opioid receptor	0.0312265582077	CHEMBL236
Kappa Opioid receptor	0.0312265582077	CHEMBL237
MEBP		
Neuronal acetylcholine receptor protein alpha-7 subunit	0.0626219668353	CHEMBL2492
Dopamine transporter (by homology)	0.0535560755162	CHEMBL238
Serotonin transporter	0.0535560755162	CHEMBL228
Norepinephrine transporter	0.0535560755162	CHEMBL222
Butyrylcholinesterase	0.0535560755162	CHEMBL1914
Calcium-activated potassium channel subunit alpha-1	0.0535560755162	CHEMBL4304
Muscarinic acetylcholine receptor M4	0.0535560755162	CHEMBL1821
Muscarinic acetylcholine receptor M2	0.0535560755162	CHEMBL211
Muscarinic acetylcholine receptor M1	0.0535560755162	CHEMBL216
Muscarinic acetylcholine receptor M3	0.0535560755162	CHEMBL245

**Table 7:** The summary of the most probable macromolecular targets for the compounds under study (PASS Targets).

Protein	Confidence	CHEMBL ID
MEP		
Mitogen-activated protein kinase 2	0.9067	CHEMBL5914
Receptor-interacting serine/threonine-protein kinase 4	0.8996	CHEMBL6083
Serine/threonine-protein kinase TNNI3K	0.8883	CHEMBL5260
G protein-coupled receptor kinase 4	0.8847	CHEMBL5861
Serine/threonine-protein kinase MRCK gamma	0.8825	CHEMBL5615
Cytochrome P450 2J2	0.8796	CHEMBL3491
Mitogen-activated protein kinase kinase kinase 3	0.8738	CHEMBL5970
Receptor tyrosine-protein kinase erbB-3	0.8062	CHEMBL5838
Homeodomain-interacting protein kinase 4	0.7941	CHEMBL1075167
Serine/threonine-protein kinase PAK 2	0.7799	CHEMBL4487
Serine/threonine-protein kinase SBK1	0.7459	CHEMBL1163129
Non-receptor tyrosine-protein kinase TNK1	0.7259	CHEMBL5334
Phosphatidylinositol-5-phosphate 4-kinase type-2 gamma	0.7252	CHEMBL1770034
Dual specificity mitogen-activated protein kinase kinase 5	0.7208	CHEMBL4948
Myotonin-protein kinase	0.7085	CHEMBL5320
Serine/threonine-protein kinase SIK2	0.7003	CHEMBL5699
Chaperone activity of bc1 complex-like, mitochondrial	0.6945	CHEMBL5550
Eukaryotic translation initiation factor 2-alpha kinase 4	0.6845	CHEMBL5358
myosin light chain kinase 2	0.6843	CHEMBL2777
Citron Rho-interacting kinase	0.6825	CHEMBL5579
Leukocyte tyrosine kinase receptor	0.6690	CHEMBL5627
Uncharacterized aarF domain-containing protein kinase 4	0.6620	CHEMBL5753
Ephrin type-A receptor 6	0.6481	CHEMBL4526
Adaptor-associated kinase	0.6397	CHEMBL3830
BMP-2-inducible protein kinase	0.6356	CHEMBL4522
Cytochrome P450 2B6	0.6337	CHEMBL4729
Serine/threonine-protein kinase SRPK3	0.6272	CHEMBL5415
Serine/threonine-protein kinase 2	0.6263	CHEMBL4202
Phosphatidylinositol-4-phosphate 5-kinase type-1 gamma	0.6195	CHEMBL1908383
Ephrin type-B receptor 6	0.5917	CHEMBL5836
Tyrosine-protein kinase CSK	0.5881	CHEMBL2634
Serine/threonine-protein kinase 32A	0.5826	CHEMBL6150
Serine/threonine-protein kinase 36	0.5790	CHEMBL4312
Tyrosine-protein kinase receptor Tie-1	0.5712	CHEMBL5274
Serine/threonine-protein kinase OSR1	0.5647	CHEMBL1163104
Serine/threonine-protein kinase PAK7	0.5536	CHEMBL4524
Peripheral plasma membrane protein CASK	0.5385	CHEMBL1908381
Serine/threonine-protein kinase NEK9	0.5364	CHEMBL5257
Ephrin type-A receptor 8	0.5318	CHEMBL4134
Estrogen receptor beta	0.5299	CHEMBL242
Serine/threonine-protein kinase PAK6	0.5149	CHEMBL4311
Ribosomal protein S6 kinase alpha 4	0.5134	CHEMBL3125
Serine/threonine-protein kinase GAK	0.5085	CHEMBL4355
prosidol		
Cytochrome P450 2J2	0.6717	CHEMBL3491
Alpha-2b adrenergic receptor	0.3277	CHEMBL1942
Muscarinic acetylcholine receptor M4	0.2869	CHEMBL1821
Muscarinic acetylcholine receptor M1	0.2819	CHEMBL216
HERG	0.2806	CHEMBL240
Protein kinase C iota	0.2505	CHEMBL2598
Histamine H1 receptor	0.2256	CHEMBL231
kazcaine		
Cytochrome P450 2J2	0.7927	CHEMBL3491
Receptor-interacting serine/threonine-protein kinase 4	0.7786	CHEMBL6083
Serine/threonine-protein kinase MRCK gamma	0.7107	CHEMBL5615
G protein-coupled receptor kinase 4	0.7034	CHEMBL5861
Mitogen-activated protein kinase kinase kinase 2	0.6794	CHEMBL5914
Mitogen-activated protein kinase kinase kinase 3	0.6458	CHEMBL5970
Serine/threonine-protein kinase SBK1	0.6177	CHEMBL1163129
Receptor tyrosine-protein kinase erbB-3	0.4501	CHEMBL5838
Serine/threonine-protein kinase SIK2	0.4465	CHEMBL5699
Eukaryotic translation initiation factor 2-alpha kinase 4	0.4396	CHEMBL5358
P-glycoprotein 1	0.4321	CHEMBL4302
Non-receptor tyrosine-protein kinase TNK1	0.4268	CHEMBL5334
myosin light chain kinase 2	0.4165	CHEMBL2777
Cytochrome P450 2B6	0.4009	CHEMBL4729
Chaperone activity of bc1 complex-like, mitochondrial	0.3753	CHEMBL5550
Serine/threonine-protein kinase TNNI3K	0.3750	CHEMBL5260
Homeodomain-interacting protein kinase 4	0.3685	CHEMBL1075167
Ephrin type-A receptor 6	0.3551	CHEMBL4526
Citron Rho-interacting kinase	0.3370	CHEMBL5579
Cytochrome P450 2C9	0.3326	CHEMBL3397
Phosphatidylinositol-5-phosphate 4-kinase type-2 gamma	0.3296	CHEMBL1770034
Plectin	0.2866	CHEMBL1293240
Protein kinase C iota	0.2864	CHEMBL2598
Leukocyte tyrosine kinase receptor	0.2831	CHEMBL5627
Estrogen receptor beta	0.2721	CHEMBL242
Muscarinic acetylcholine receptor M4	0.2553	CHEMBL1821
Myotonin-protein kinase	0.2550	CHEMBL5320
Ephrin type-B receptor 6	0.2545	CHEMBL5836
AEPP		
Cytochrome P450 2J2	0.6780	CHEMBL3491
P-glycoprotein 1	0.4892	CHEMBL4302
Alpha-2b adrenergic receptor	0.3261	CHEMBL1942
Cytochrome P450 2D6	0.3077	CHEMBL289
Protein kinase C iota	0.2962	CHEMBL2598
Muscarinic acetylcholine receptor M1	0.2911	CHEMBL216
Muscarinic acetylcholine receptor M4	0.2855	CHEMBL1821
Histamine H1 receptor	0.2280	CHEMBL231
BVBP		
Serine/threonine-protein kinase 35	0.3745	CHEMBL5651
Cytochrome P450 2J2	0.3266	CHEMBL3491
Plectin	0.2873	CHEMBL1293240
Muscarinic acetylcholine receptor M2	0.2740	CHEMBL211
Receptor tyrosine-protein kinase erbB-3	0.2663	CHEMBL5838
Mitogen-activated protein kinase kinase kinase 3	0.2531	CHEMBL5970
Serine/threonine-protein kinase SIK3	0.2523	CHEMBL6149
BBB		
P-glycoprotein 1	0.5329	CHEMBL4302
Cytochrome P450 2J2	0.3725	CHEMBL3491
Serine/threonine-protein kinase 35	0.3521	CHEMBL5651
Muscarinic acetylcholine receptor M5	0.3343	CHEMBL2035
Microtubule-associated serine/threonine-protein kinase 1	0.3154	CHEMBL1163128
Mitogen-activated protein kinase 6	0.3104	CHEMBL5121
Serine/threonine-protein kinase PFTAIRE-1	0.3071	CHEMBL6162
Protein kinase C alpha	0.2654	CHEMBL299
Muscarinic acetylcholine receptor M4	0.2492	CHEMBL1821
Serotonin 3a (5-HT3a) receptor	0.2476	CHEMBL1899
EEHP		
Cytochrome P450 2J2	0.8460	CHEMBL3491
Receptor-interacting serine/threonine-protein kinase 4	0.8042	CHEMBL6083
Serine/threonine-protein kinase MRCK gamma	0.8001	CHEMBL5615
Mitogen-activated protein kinase kinase kinase 2	0.7452	CHEMBL5914
G protein-coupled receptor kinase 4	0.7240	CHEMBL5861
Mitogen-activated protein kinase kinase kinase 3	0.7050	CHEMBL5970
Serine/threonine-protein kinase PAK 2	0.6813	CHEMBL4487
Serine/threonine-protein kinase SBK1	0.6791	CHEMBL1163129
Receptor tyrosine-protein kinase erbB-3	0.6538	CHEMBL5838
Serine/threonine-protein kinase TNNI3K	0.6324	CHEMBL5260
Chaperone activity of bc1 complex-like, mitochondrial	0.5796	CHEMBL5550
Cytochrome P450 2B6	0.5447	CHEMBL4729
Eukaryotic translation initiation factor 2-alpha kinase 4	0.5391	CHEMBL5358
Serine/threonine-protein kinase SIK2	0.5357	CHEMBL5699
Estrogen receptor beta	0.5281	CHEMBL242
Citron Rho-interacting kinase	0.5175	CHEMBL5579
MEBP		
Receptor-interacting serine/threonine-protein kinase 4	0.8715	CHEMBL6083
Mitogen-activated protein kinase kinase kinase 2	0.8575	CHEMBL5914
G protein-coupled receptor kinase 4	0.8548	CHEMBL5861
Serine/threonine-protein kinase MRCK gamma	0.8136	CHEMBL5615
Cytochrome P450 2J2	0.8101	CHEMBL3491
Mitogen-activated protein kinase kinase kinase 3	0.7846	CHEMBL5970
Serine/threonine-protein kinase TNNI3K	0.7075	CHEMBL5260
Homeodomain-interacting protein kinase 4	0.7053	CHEMBL1075167
Non-receptor tyrosine-protein kinase TNK1	0.6899	CHEMBL5334
Serine/threonine-protein kinase SBK1	0.6805	CHEMBL1163129
Phosphatidylinositol-5-phosphate 4-kinase type-2 gamma	0.6226	CHEMBL1770034
myosin light chain kinase 2	0.6214	CHEMBL2777
Serine/threonine-protein kinase SIK2	0.6165	CHEMBL5699
Eukaryotic translation initiation factor 2-alpha kinase 4	0.5802	CHEMBL5358
Leukocyte tyrosine kinase receptor	0.5779	CHEMBL5627
Receptor tyrosine-protein kinase erbB-3	0.5648	CHEMBL5838
Tyrosine-protein kinase receptor Tie-1	0.5502	CHEMBL5274
Ephrin type-A receptor 6	0.5471	CHEMBL4526
BMP-2-inducible protein kinase	0.5423	CHEMBL4522
Adaptor-associated kinase	0.5418	CHEMBL3830
Chaperone activity of bc1 complex-like, mitochondrial	0.5405	CHEMBL5550
Cytochrome P450 2B6	0.5163	CHEMBL4729
Myotonin-protein kinase	0.5140	CHEMBL5320

**Table 8:** The results of the predicted acute toxicity for the studied compounds.

Rat LD_50_ for Different Routes of Administration *	Meaning/Acceptability **						
	MEP	Prosidol	Kazcaine	AEPP	BVBP	EEHP	MEBP
**IP** (mg/kg)	**92.5**	**206.5**	**343.5**	**229.3**	**362.8**	**122.3**	**245.2**
**IP** (log10, mmol/kg)	**−0.190**	**−0.170**	**-**	**−0.104**	**−0.008**	**−0.208**	**0.003**
**IP** acute toxic class	**4**	**4**	4	**4**	**4**	**4**	*4*
**IV** (mg/kg)	**27.91**	**33.25**	343.50	**31.73**	**26.55**	**28.52**	**23.67**
**IV** (log10, mmol/kg)	**−0.710**	**−0.963**	-	**−0.963**	**−1.144**	**0.389**	**−1.012**
**IV** acute toxic class	**3**	**3**	5	**3**	**3**	**3**	**3**
**Oral** (mg/kg)	*332.3*	**557.5**	343.5	**570.4**	**881.7**	**483.7**	**439.0**
**Oral** (log10, mmol/kg)	*0.365*	**0.261**	-	**0.292**	**0.378**	**0.389**	**0.256**
**Oral** acute toxic class	*4*	**4**	4	**4**	**4**	**4**	**4**
**SC** (mg/kg)	**105.0**	**275.0**	343.5	**235.1**	*265.1*	**273.0**	**394.8**
**SC** LD50						**0.141**	**0.210**
**SC** acute toxic class	**3**	**4**	4	**4**	*4*	**4**	**4**

* IP—intraperitoneal route of administration, IV—intravenous route of administration, Oral—oral route of administration, and SC—subcutaneous route of administration. ** BOLD *=* in AD: the compound falls within the range of applicability of the models; italic = out of AD: compound outside the range of applicability of models.

**Table 9:** The prognosis of adverse effects for the compounds under study.

Compound	Pa *	Pi *	P	Adverse Effect
MEP	0.784	0.066	0.718	hepatotoxicity
prosidol	0.416	0.172	0.244	arrhythmia
kazcaine	0.306	0.295	0.011	arrhythmia
	0.729	0.089	0.640	hepatotoxicity
	0.276	0.258	0.018	myocardial infarction
	0.264	0.197	0.067	nephrotoxicity
AEPP	0.439	0.156	0.283	arrhythmia
	0.333	0.318	0.015	hepatotoxicity
BVBP	0.571	0.060	0.511	arrhythmia
BBB **	0.678	0.029	0.649	arrhythmia
EEHP	0.729	0.089	0.640	hepatotoxicity
	0.263	0.198	0.065	nephrotoxicity
MEBP	0.788	0.064	0.724	hepatotoxicity
	0.309	0.180	0.129	myocardial infarction

* Pa—probability of activity; Pi—probability of inactivity. ** only as a base.

**Table 10:** The studied compounds and their host–guest complexes with β-CD.

#	Molecule Name	R	Guest/β-CD	Mass Guest/Host/Complex	Included Part of the Guest
1	MEP	H	1/1	139/1135/1274	Full inclusion
2	kazcaine	COC_6_H_5_	1/2	301/2270/2571	1-2-ethoxyethyl and piperidine
3	AEPP	CH_3_	1/2	291/2270/2561	1-(2-ethoxyethyl)-4-phenylpiperidine
	prosidol	CH_2_CH_3_	1/2	305/2270/2575	Full inclusion
4	BBB·HCl	H	1/2	319/2270/2589	1-(3-n-butoxypropyl) and 4-benzoyloxy
	BVBP	CCCHCH_2_	1/2	370/2270/2640	1-(3-n-butoxypropyl) and 4-benzoyloxy-piperidine

**Table 11:** The local anesthetic activity of the compounds and reference drugs for the infiltration anesthesia, using the Bulbring-Wade method.

Compound (Code), Concentration, %	Anesthesia Index (M ± m)	The Duration of Complete Anesthesia (min.), (M ± m)	Total Duration of the Effect (min.), (M ± m)	Ref.
kazcaine, 1%	-	67.4 ± 1.9	101.9 ± 3.5	[7]
kazcaine: β-CD (1:2), 1%	-	121.3 ± 4.3 *^a^*	136.1 ± 1.7 *^a^*	[7]
kazcaine, 0.5%	-	26.3 ± 2.9	82.9 ± 3.6	[7]
kazcaine: β-CD (1:2) *^b^*, 0,5%	-	63.3 ± 2.9	108.4 ± 2.7	[7]
BVBP, 0.5%	36.0 ± 0	65.0 ± 0	90.0 ± 1.3	[18]
BVBP:β-CD (1:2), 0.5% (KFCD-4)	36.0 ± 0	48.0 ± 4.5	73.3 ± 2.1	[18]
BBB-HCl, 0.25%	-	23.3 ± 3.8	35.0 ± 2.7	[35]
BBB-HCl, 0.5%	-	94.2 ± 1.5	102.5 ± 2.1	[35]
BBB-HCl: β-CD (1:2), 0.5%	35.0 ± 1.4	40.0 ± 2.6 *^d^*	93.3 ± 3.1 *^c^*	[18]
AEPP:β-CD (1:2), 0.5% (KFCD-6)	36.0 ± 1.3	59.0 ± 2.7	87.1 ± 4.2	[20]
MEP: β-CD, 0.5% (KFCD-7)	35.4 ± 1.3	33.3 ± 1.6	47.8 ± 3.6	This work
procaine, 1%	-	20.1 ± 1.6	42.0 ± 1.2	[7]
trimecaine, 0.5%	34.1 ± 0.5	30.0 ± 1.7	54.5 ± 2.3	[20]
lidocaine, 0.5%	32.3 ± 2.3	25.8 ± 0.8	44.1 ± 1.7	[20]
procaine, 0.5%	30.0 ± 0.2	10.0 ± 0	22.0 ± 0.1	[20]

*^a^* Deviations in relation to reference preparations are statistically authentic at *p* < 0.001. *^b^* By mass kazcaine is 1/10 of the complex. *^c^* Deviations in relation to the reference preparations are statistically authentic at: lidocaine—p_i_ < 0.05, trimecaine—p_i_ < 0.001, procaine—p_i_ < 0.02. *^d^* statistically authentic at: lidocaine—p_i_ < 0.05, trimecaine—p_i_ < 0.01, procaine—p_i_ < 0.001.

**Table 12:** The local anesthetic activity of compounds and reference drugs for the conduction anesthesia.

Compound (Code)	The Duration of the Complete Anesthesia (min.), (M ± m)		The Total Duration of Effect (min.), (M ± m)		Ref.
concentration	0.5%	1%	0.5%	1%	
kazcaine	74.4 ± 11.1	103.4 ± 11.1	97.6 ± 6.3	119.6 ± 5.5	[7]
kazcaine: β-CD (1:2)	106.1 ± 2.0 *^a^*	137.1 ± 3.9 *^b^*	118.9 ± 6.8 *^a^*	147.5 ± 6.7 *^a^*	[7]
BVBP	60	180	-	-	
BVBP:β-CD (1:2) KFCD-4	-	68.2 ± 6.7 *^d^*	-	80.5 ± 12.0 *^d^*	
BBB-HCl	67.5 ± 3.4 *^f^*	50.8 ± 3.0 *^e^*	86.7 ± 3.6 *^f^*	150.8 ± 4.7 *^e^*	[35]
BBB-HCl: β-CD (1:2)	62.5 ± 1.2 ^*c*,*d*^	-	83.3 ± 2.4 ^*c*,*d*^	-	[18]
AEPP:β-CD (1:2) KFCD-6	-	89.4 ± 13.4 *^d^*	-	138.5 ± 14.8 *^d^*	[20]
MEP	-	-	-	-	
MEP:β-CD (1:1) KFCD-7	-	66.2 ± 10.5 *^d^*	-	73.5 ± 11.3 *^d^*	This work
trimecaine	33.7 ± 11.2 *^d^*	46.9 ± 8.1 *^d^*	45.8 ± 13.2 *^d^*	58.1 ± 11.4 *^d^*	[18,20]
lidocaine	28.0 ± 5.4 *^d^*	52.7 ± 6.4 *^d^*	45.0 ± 4.7 *^d^*	63.1 ± 16.2 *^d^*	[18,20]
procaine	15.2 ± 3.9 *^d^*	34.2 ± 6.9 *^d^*	30.4 ± 4.2 *^d^*	41.3 ± 14.6 *^d^*	[18,20]

*^a^* Deviations in relation to reference preparations are statistically authentic at *p* < 0.001. *^b^* Deviations in relation to kazcaine are statistically authentic at *p* < 0.01. *^c^* Deviations in relation to reference preparations are statistically authentic at: lidocaine—p_i_ < 0.05, trimecaine—p_i_ < 0.001, procaine—p_i_ < 0.02. *^d^* rate of anesthesia induction—3 min. *^e^* Local anesthetic activity for the conduction anesthesia, using the method of electrical stimulation of a rabbit inferior dental nerve. *^f^* Local anesthetic activity for the conduction anesthesia, using a modified “tail flick” method.

**Table 13:** The acute toxicity of the compounds under study and the reference drugs.

Compound	LD_50_ (mg/kg)	*p*	Ref.
kazcaine	529.3 ± 7.1		[7]
kazcaine: β-CD (1:2) *	590.0 ± 11.3		[7]
BVBP	316		[37]
BVBP: β-CD (1:2) (KFCD-4)	700.0 ± 25.4		[18]
BBB-HCl	138		[35]
BBB-HCl: β-CD (1:2)	478.5 ± 8.0		[18]
AEPP	340		[37]
AEPP:β-CD (1:2) (KFCD-6) **	830.0 ± 34.5		[20]
MEP:β-CD (KFCD-7) **	622.4 ± 22.9		This work
procaine	480.0 ± 9.8	p_1_	[18]
lidocaine	248.6 ± 18.4	p_2_	[18]
trimecaine	378.2 ± 19.4	p_3_	[7]

* Deviations for this complex compared to the reference preparations are statistically authentic at p_i_ < 0.001. ** Deviations for the KFCD-6 and KFCD-7 compared to the reference preparations are statistically authentic at p_1_ < 0.01; p_2_ and p_3_ < 0.005.

## Data Availability

Data are contained within the article and Supplementary Materials.

## Supplementary Materials

The following supporting information can be downloaded at https://www.mdpi.com/article/10.3390/molecules29051098/s1, Table S1: The predicted biological activity for the studied compounds.
