# Supplementary material for: Comparative Analysis of the Structure and Pharmacological Properties of Some Piperidines and Host–Guest Complexes of β-Cyclodextrin
Source: Molecules. 2024 Feb 29;29(5):1098. doi: 10.3390/molecules29051098 (PMC10935227; doi:10.3390/molecules29051098)
Supplement: Supplementary file 1 [file molecules-29-01098-s001.zip › molecules-2897354-supplementary.pdf]

Supplementary Materials

Table S1. Predicted biological activity for the studied compounds.

Pa	***Pi***	***F***	***Biological activity***
**$C_{18}H_{27}NO_3$ [1-(2-ethoxyethyl)-4-phenyl-4-propionyl-oxypiperidine]**			
0.756	0.004	0.752	Anesthetic
0.735	0.003	0.732	Anesthetic local
0.694	0.008	0.686	Antipruritic
0.683	0.004	0.679	Antitussive
0.646	0.014	0.632	Antisecretoric
0.624	0.020	0.604	Spasmolytic, urinary
0.607	0.011	0.596	Antipruritic, allergic
0.608	0.019	0.589	Analgesic
0.650	0.064	0.586	Antieczematic
0.591	0.015	0.576	Spasmolytic
0.566	0.024	0.542	Anticonvulsant
0.539	0.004	0.535	Antiparkinsonian, tremor relieving
0.523	0.010	0.513	Spasmolytic, Papaverin-like
0.474	0.035	0.439	Analeptic
0.489	0.067	0.422	Antidyskinetic
0.366	0.039	0.327	Skeletal muscle relaxant
0.448	0.145	0.303	Antiischemic, cerebral
0.388	0.086	0.302	Antihypoxic
0.362	0.064	0.298	Glyoxylate reductase inhibitor
0.320	0.032	0.288	Sclerosant
0.377	0.091	0.286	Radiosensitizer
0.301	0.049	0.252	Antiinfertility, female
0.341	0.089	0.252	Vasodilator, coronary
0.341	0.096	0.245	Antimyopathies
0.331	0.125	0.206	Vasoprotector
0.252	0.101	0.151	Immunomodulator
0.187	0.062	0.125	Antimycoplasmal
0.159	0.034	0.125	Cholinergic antagonist
0.196	0.075	0.121	Antiosteoporotic
0.224	0.115	0.109	Immunostimulant
0.157	0.057	0.100	Histamine agonist
0.260	0.166	0.094	Antinephritic
0.226	0.140	0.086	Cardiotonic
0.220	0.153	0.067	Aspergillopepsin I inhibitor
0.164	0.100	0.064	Antianorexic
0.094	0.039	0.055	Antidote, heavy metal
0.096	0.050	0.046	Beta-D-fucosidase inhibitor

Pa	*Pi*	*F*	*Biological activity*
0.117	0.071	0.046	Sulfate adenylyltransferase (ADP) inhibitor
0.099	0.053	0.046	Alcohol oxidase inhibitor
0.124	0.100	0.024	Antihistaminic
0.142	0.129	0.013	Antipsychotic
0.195	0.181	0.014	Urethanase inhibitor
0.104	0.093	0.011	Anabolic
0.041	0.033	0.008	Chelator, Iron
0.163	0.158	0.005	Antialcoholic
**$C_{18}H_{23}NO_3$ [1-(2-ethoxyethyl)-4-ethynyl-4-benzoyloxypiperidine]**			
0.736	0.007	0.729	Antisecretoric
0.711	0.004	0.707	Anesthetic
0.689	0.004	0.685	Spasmolytic. Papaverin-like
0.682	0.012	0.670	Spasmolytic. urinary
0.710	0.042	0.668	Antieczematic
0.616	0.004	0.612	Anesthetic local
0.586	0.016	0.570	Spasmolytic
0.605	0.070	0.535	Fibrinolytic
0.552	0.026	0.526	Anticonvulsant
0.523	0.009	0.514	Antitussive
0.527	0.044	0.483	Phosphatidylcholine-retinol O-acyltransferase inhibitor
0.520	0.038	0.482	Antianginal
0.481	0.048	0.433	Antipruritic. allergic
0.477	0.071	0.406	Ovulation inhibitor
0.407	0.022	0.385	Antiosteoporotic
0.429	0.047	0.382	Dermatologic
0.415	0.052	0.363	Analeptic
0.389	0.032	0.357	Muscle relaxant
0.313	0.029	0.284	Antiperistaltic
0.344	0.073	0.271	Antiallergic
0.325	0.053	0.272	Skeletal muscle relaxant
0.299	0.052	0.247	Antiischemic
0.331	0.088	0.243	Antipruritic
0.297	0.145	0.152	Antimyopathies
0.237	0.092	0.145	Antialcoholic
0.122	0.054	0.068	Anabolic
**$C_{17}H_{25}NO_3$ [4-acetoxy-1-(2-ethoxyethyl)-4-phenylpiperidine]**			
0.830	0.003	0.827	Antitussive
0.762	0.004	0.758	Anesthetic
0.736	0.003	0.733	Anesthetic local
0.748	0.055	0.693	Phobic disorders treatment
0.682	0.009	0.673	Spasmolytic

Pa	*Pi*	*F*	*Biological activity*
0.673	0.004	0.669	Gonadotropin antagonist
0.688	0.027	0.661	Fibrinolytic
0.637	0.003	0.634	Antiparkinsonian. tremor relieving
0.638	0.014	0.624	Lysostaphin inhibitor
0.633	0.019	0.614	Spasmolytic. urinary
0.621	0.077	0.544	Antieczematic
0.610	0.015	0.595	Antipruritic
0.583	0.021	0.562	Analeptic
0.576	0.023	0.553	Analgesic
0.559	0.020	0.539	Antipruritic. allergic
0.539	0.028	0.511	Anticonvulsant
0.503	0.029	0.474	Antisecretoric
0.513	0.060	0.453	Antidyskinetic
0.467	0.015	0.452	Spasmolytic. Papaverin-like
0.451	0.004	0.447	Antiperistaltic
0.469	0.056	0.413	Antinociceptive
0.399	0.011	0.388	Antinaupathic
0.426	0.072	0.354	Fibrinogen receptor antagonist
0.373	0.024	0.349	Antiinfertility. female
0.332	0.077	0.255	Leukopoiesis inhibitor
0.297	0.046	0.251	Antipyretic
0.287	0.043	0.244	Sclerosant
0.317	0.077	0.24	Radioprotector
0.331	0.095	0.236	Sphinganine kinase inhibitor
0.282	0.046	0.236	Oryzin inhibitor
0.311	0.083	0.228	Antiasthmatic
0.331	0.106	0.225	Antimyopathies
0.307	0.095	0.212	Cyanoalanine nitrilase inhibitor
0.263	0.056	0.207	Pediculicide
0.340	0.137	0.203	Antianginal
0.322	0.133	0.189	Vasoprotector
0.268	0.082	0.186	Skeletal muscle relaxant
0.245	0.060	0.185	Antiepileptic
0.259	0.075	0.184	Muscle relaxant
0.359	0.192	0.167	Antineurotic
0.254	0.087	0.167	Ophthalmic drug
0.182	0.083	0.099	Phosphatidylglycerophosphatase inhibitor
0.184	0.085	0.099	Mucolytic
0.174	0.075	0.099	Acaricide
0.149	0.051	0.098	Glycerone-phosphate O-acyltransferase inhibitor
0.163	0.066	0.097	Leishmanolysin inhibitor
0.299	0.201	0.098	Gastrin inhibitor

Pa	*Pi*	*F*	*Biological activity*
0.162	0.107	0.055	Antiuremic
**$C_{23}H_{31}NO_3$ [1-(3-n-Butoxypropyl)-4-vinylacetylene-4-benzoyloxypiperidine]**			
0.782	0.022	0.760	Antieczematic
0.741	0.004	0.737	Anesthetic
0.733	0.003	0.730	Anesthetic local
0.581	0.007	0.574	Spasmolytic, Papaverin-like
0.570	0.020	0.550	Dermatologic
0.558	0.020	0.538	Antipruritic, allergic
0.507	0.033	0.474	Antipruritic
0.475	0.033	0.442	Spasmolytic
0.474	0.049	0.425	Antianginal
0.458	0.061	0.397	Spasmolytic, urinary
0.429	0.048	0.381	Antisecretoric
0.380	0.048	0.332	Antipsoriatic
0.465	0.134	0.331	Fibrinolytic
0.328	0.024	0.304	Antiperistaltic
0.321	0.098	0.223	Analeptic
0.263	0.077	0.186	Antiischemic
0.226	0.074	0.152	Sclerosant
0.226	0.095	0.131	Muscle relaxant
0.294	0.164	0.130	Chemosensitizer
0.281	0.152	0.129	Radiosensitizer
0.185	0.078	0.107	Antinaupathic
0.241	0.159	0.082	Anticonvulsant
0.203	0.136	0.067	Skeletal muscle relaxant
0.124	0.066	0.058	Antiemetic
**$C_{19}H_{29}NO_3$ [1-(3-n-butoxypropyl)-4-benzoyloxypiperidin hydrochloride][1]**			
0.899	0.002		Anesthetic local
0.896	0.003		Anesthetic
0.848	0.003		Spasmolytic, Papaverin-like
0.832	0.004		Spasmolytic
0.835	0.010		G-protein-coupled receptor kinase inhibitor
0.835	0.010		Beta-adrenergic receptor kinase inhibitor
0.788	0.019		Alkenylglycerophosphocholine hydrolase inhibitor
0.727	0.014		Fibrinolytic
0.734	0.035		Antieczematic
0.707	0.009		Antisecretoric
0.684	0.012		Spasmolytic, urinary
0.671	0.003		Antiparkinsonian. tremor relieving

[1] PASS Online does not count it in the form of hydrochloride, but does it in a base form.

Pa	*Pi*	*F*	*Biological activity*
0.626	0.009		Antipruritic. allergic
0.551	0.022		Antithrombotic
0.532	0.027		Analeptic
0.553	0.099		Nootropic
0.470	0.025		Anesthetic general
0.469	0.074		Anaphylatoxin receptor antagonist
0.416	0.034		Vasodilator
0.396	0.031		Muscle relaxant
0.482	0.118		Antineurotic
0.376	0.013		Antinaupathic
0.367	0.010		Antihistaminic
0.340	0.020		Antiperistaltic
0.394	0.090		Antianginal
0.341	0.050		Immunomodulator
0.316	0.045		Ophthalmic drug
**$C_{11}H_{19}NO_2$ [1-(2-ethoxyethyl)-4-ethynyl-4-hydroxypiperidine]**			
0.815	0.003	0.812	Gonadotropin antagonist
0.745	0.006	0.739	Antisecretoric
0.747	0.013	0.734	Aldehyde oxidase inhibitor
0.700	0.027	0.673	Mannotetraose 2-alpha-N-acetylglucosaminyltransferase inhibitor
0.662	0.005	0.657	Gestagen antagonist
0.660	0.012	0.648	Anticonvulsant
0.651	0.007	0.644	Antiosteoporotic
0.635	0.019	0.616	Spasmolytic, urinary
0.649	0.065	0.584	Antieczematic
0.547	0.029	0.518	Respiratory analeptic
0.530	0.028	0.502	Antipruritic, allergic
0.525	0.028	0.497	Analeptic
0.426	0.016	0.41	Anesthetic
0.406	0.048	0.358	Spasmolytic
0.403	0.058	0.345	Antipruritic
0.355	0.020	0.335	Antiparkinsonian, tremor relieving
0.340	0.013	0.327	Anesthetic local
0.333	0.022	0.311	Antiperistaltic
**$C_{15}H_{17}NO_2$ [1-methyl-4-ethynyl-4- benzoyloxypiperidine]**			
0.695	0.006	0.689	Ovulation inhibitor
0.655	0.013	0.642	Antisecretoric
0.626	0.006	0.62	Gestagen antagonist
0.583	0.021	0.562	Anticonvulsant
0.593	0.041	0.552	Anaphylatoxin receptor antagonist
0.476	0.004	0.472	Antiemetic
0.497	0.050	0.447	Spasmolytic, urinary

Pa	*Pi*	*F*	*Biological activity*
0.441	0.014	0.427	Anesthetic
0.475	0.050	0.425	Neurotransmitter antagonist
0.510	0.091	0.419	Kidney function stimulant
0.430	0.057	0.373	Respiratory analeptic
0.396	0.022	0.374	Monoamine uptake inhibitor
0.413	0.046	0.367	Spasmolytic
0.497	0.138	0.359	Antieczematic
0.363	0.011	0.352	Anesthetic local
0.372	0.035	0.337	Loop diuretic
0.422	0.086	0.336	Glutamate-5-semialdehyde dehydrogenase inhibitor
0.421	0.092	0.329	Antinociceptive
0.363	0.038	0.325	Antialcoholic
0.338	0.020	0.318	Antiperistaltic
0.351	0.042	0.309	Muscle relaxant
0.358	0.052	0.306	Anesthetic general
0.325	0.024	0.301	Vascular dementia treatment
0.342	0.046	0.296	Glucan 1,4-alpha-maltotetraohydrolase inhibitor
0.352	0.061	0.291	Muscular dystrophy treatment
0.353	0.067	0.286	Antiinfective
0.329	0.056	0.273	Diuretic inhibitor
0.348	0.084	0.264	Analeptic
0.246	0.090	0.156	Antiparkinsonian, rigidity relieving
